# Interplay between Interferon-Mediated Innate Immunity and Porcine Reproductive and Respiratory Syndrome Virus

**DOI:** 10.3390/v4040424

**Published:** 2012-04-02

**Authors:** Yan Sun, Mingyuan Han, Chiyong Kim, Jay G. Calvert, Dongwan Yoo

**Affiliations:** 1 Department of Pathobiology, University of Illinois at Urbana-Champaign, Urbana, IL 61802, USA; Email: yansun3@illinois.edu (Y.S.); mhan10@illinois.edu (M.H.); ckim102@illinois.edu (C.K.); 2 Pfizer Animal Health, Kalamazoo, MI 49007, USA; Email: jay.calvert@pfizer.com

**Keywords:** arterivirus, interferon, PRRS, PRRSV, RIG-I, MDA5, NF-κB, JAK-STAT, non-structural proteins, Nsp, nucleocapsid

## Abstract

Innate immunity is the first line of defense against viral infection, and in turn, viruses have evolved to evade host immune surveillance. As a result, viruses may persist in host and develop chronic infections. Type I interferons (IFN-α/β) are among the most potent antiviral cytokines triggered by viral infections. Porcine reproductive and respiratory syndrome (PRRS) is a disease of pigs that is characterized by negligible induction of type I IFNs and viral persistence for an extended period. For IFN production, RIG-I/MDA5 and JAK-STAT pathways are two major signaling pathways, and recent studies indicate that PRRS virus is armed to modulate type I IFN responses during infection. This review describes the viral strategies for modulation of type I IFN responses. At least three non–structural proteins (Nsp1, Nsp2, and Nsp11) and a structural protein (N nucleocapsid protein) have been identified and characterized to play roles in the IFN suppression and NF-κB pathways. Nsp’s are early proteins while N is a late protein, suggesting that additional signaling pathways may be involved in addition to the IFN pathway. The understanding of molecular bases for virus-mediated modulation of host innate immune signaling will help us design new generation vaccines and control PRRS.

## 1. Introduction

Porcine reproductive and respiratory syndrome (PRRS) is one of the most economically important diseases affecting the swine industry worldwide. The disease was first observed in the USA in 1987 and then in Germany in 1990 [1]. The clinical signs include reproductive failures, including late-term abortions, increased number of stillborn and mummified fetuses in sows, and respiratory problems in pigs of all ages. The etiology of PRRS was first determined in 1990 in the Netherlands and named Lelystad virus (LV) [1]. Shortly after, porcine reproductive and respiratory syndrome virus (PRRSV) VR2332 was isolated in the USA and shown to be the sole causative agent for PRRS [1,2]. Comparative genomic sequence analyses reveal the genetic differences between LV and VR2332 and accordingly PRRS viruses are grouped into two genotypes; European (Type I) and North American (Type II) types [3,4]. A highly pathogenic PRRSV (HP-PRRSV) emerged in China in 2006 and was thought to be a mutant belonging to the North American type. Clinical studies show that the affected animals develop high fever, depression, anorexia, dyspnoea, reddening of the skin, edema of the eyelids, mild diarrhea, shivering, limping, and unusually high morbidity (50%–100%) and mortality (20%–100%) in pigs of all ages [5].

PRRSV belongs to the genus Arterivirus which is placed in the family Arteriviridae together with equine arteritis virus (EAV), lactate dehydrogenase-elevating virus of mice (LDV), and simian hemorrhagic fever virus (SHFV). The Arteriviridae family, along with the Coronaviridae and Roniviridae families, comprise the order Nidovirales [6,7,8,9]. The PRRSV genome consists of a positive-sense single-stranded RNA of 15 Kb in size, encoding 10 ORFs: ORF1a, ORF1b, ORF2a, ORF2b, ORFs 3 through 7, plus the newly identified ORF5a [10,11]. ORF1a and ORF1b code for two large polyproteins, pp1a and pp1a/b, with expression of the latter by ribosomal frame-shifting [12,13]. Pp1a and pp1a/b are proteolytically processed into 14 nonstructural proteins (Nsp’s) by the papain-like cysteine protease (PCP) in Nsp1, poliovirus 3C-like cysteine protease (CP) in Nsp2, and serine protease (SP) in Nsp4. Nsp3 through Nsp12 are cleaved by SP, and PCP generates Nsp1α and Nsp1ß, while CP generates Nsp2 [14]. ORF2a, ORF2b, ORF3 through ORF7, and ORF5a code for seven structural proteins: glycoprotein (GP) 2, small envelop (E), GP3, GP4, GP5, membrane (M), nucleocapsid (N) proteins, and the newly identified ORF5a protein [15,16]. GP5 and M form a dimer, and GP2, GP3, and GP4 form a hetero-trimer that may facilitate the virus entry [17,18]. N protein binds to the viral genome RNA and also interacts with itself to form the core capsid by homo–oligomerization during the viral particle assembly [19,20]. Some of the PRRSV proteins are multi-functional and shown to play modulatory roles for host innate immunity [14,21]. Interferons (IFN) are potent immune responsive cytokines against invading viruses, and their modulations by PRRSV have recently been studied. Although their precise mechanisms of action remain to be further elucidated, PRRSV exhibits significant inhibition of type I IFN production in both virus-infected cells and pigs. The current review summarizes the innate immune signaling pathways of host and discusses the current knowledge as to how PRRSV interferes with the type I IFN signaling pathways in particular.

## 2. Signaling Pathways for Innate Immunity against Virus Infection

**Figure 1:**
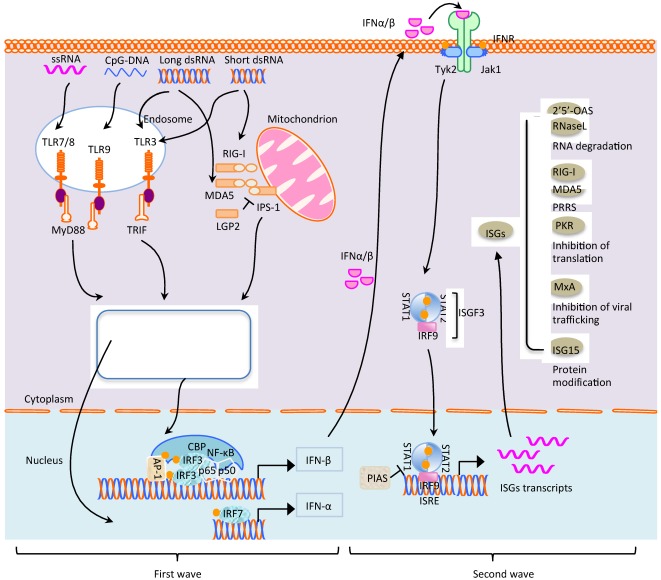
Signal pathways for type I IFN production and IFN stimulated gene expressions. Double-stranded RNA, single-stranded RNA, and CpG DNA are recognized by TLR3, TLR7/8, and TLR9, respectively, in the endosomes, which leads to the dimerization of receptors and recruitment of TRIF or MyD88. The stimulation results in the assembly of signaling complexes and initiation of signaling cascades leading to the phosphorylation and activation of IRF3/IRF7, NF-κB, and AP-1. Once activated, these transcription factors translocate to the nucleus, and together with CBP, induce the transcription of IFN-α and IFN-β. Activation of RIG-I/MDA5 by viral RNA may be inhibited by LGP2. Both RIG-I and MDA5 activates IPS-1 through the CARD domain. IPS-1 then induces signaling pathways resulting in the activation of IRF3, IRF7, and NF-κB through different adaptors and kinases. IPS-1 signaling also likely involves the MAP kinase cascade for activation of AP-1. The nuclear signal of IRF3/IRF7, NF-κB, and AP-1 is similar to that of TLR. Once IFN-α and IFN-β are secreted extracellularly outside the cell after production, they bind to IFN receptors on itself (autocrine) or neighbor cells (paracrine), the receptor associated JAK1 kinase is phosphorylated and activated to recruit STAT1 and STAT2 through their SH2 domain for phosphorylation. The phosphorylated STAT1 and STAT2 are detached from the IFN receptors and associated each other to form ISGF3 complexes along with IRF9. ISGF3 then undergoes nuclear translocation and binds to ISRE to induce the transcription of hundreds of ISGs. Five major kinds of ISGs are listed here; MxA, OAS–1/RNaseL, RIG-I/MDA5, ISG15, and PKR. For their modes of action, see the text. Grey circle indicates the lysine-48-linked ubiquitin chain which leads to the degradation of proteins. Yellow circle indicates phosphorylation.

IFNs are multifunctional cytokines that play important roles for antiviral defense and shaping adaptive immunity. IFNs are commonly classified into type I and type II. Type I IFNs are the main cytokines for innate immunity against viral infections and include various subtypes depending on the animal species. Type I IFNs in humans consists of 13 subtypes for IFN-α and a single subtype for IFN-ß, -ω, -κ, and -ε [22]. Limitin/IFN-like 1 is an additional type I IFN-like cytokine recently identified in mice that also binds the type I IFN receptor [23]. Similar to humans, IFN-α in swine is encoded by as many as 17 functional genes. The IFN-δ-like molecule is found only in pigs and cattle and designated SPI IFN [24,25]. IFN-γ is the sole representative of type II IFN. While type I IFNs are produced in most cell types in response to different viruses, type II IFN is produced by limited types of cells including natural killer (NK) cells, activated T lymphocytes, macrophages, and neurons. Type III IFNs have recently been described as containing 3 subtypes IFN-λ1, IFN-λ2, and IFN-λ3, but their role in antiviral defense remains to be established [22]. Since type I IFNs are the most notable cytokines in the fight against viral infection at an early stage, a focus will be given to discuss how they are produced in virus-infected cells and act on neighbor cells to prevent the spread of virus (Figure 1).

### 2.1. Toll-Like Receptor (TLR) Signaling

Toll-like receptors (TLRs) have been investigated extensively in the past few years and a considerable progress has been made for mammalian TLRs and their functions. TLRs are a family of proteins recognizing different pathogen-associated molecule patterns (PAMPs). The key signaling domain unique to the TLR system is the toll/interleukin (IL)-1 receptor (TIR) domain located in the cytosolic face of each TLR and also in the adapters [26]. Once engaged to TLRs, PAMPs recruit different kinases through different adaptors by the TIR domain and thus trigger different signaling pathways, leading to the expression of specific genes that are involved in the removal of invading viruses. Ten TLRs have so far been identified for humans and 11 TLRs for mice [27]. Of these, TLRs 3, 7, 8, and 9 are discussed in this review since they contribute the most to the control of virus infection [28]. 

#### 2.1.1. TLR3

Most cell types express TLR3 in the intracellular endosomes, except human fibroblast in which TLR3 is found to act on the cell surface [29]. TLR3 mediates the response to both poly(I:C) and dsRNA [30,31]. After stimulation, the TIR domain of TLR3 binds to the TIR-containing adaptor protein inducing IFN-ß (TRIF) which indirectly activates several transcription factors including NF (nuclear factor)-κB, IRF (interferon regulation factor) 3, and AP (activating protein)-1 [32]. To mediate IRF3 activation, the N-terminal domain of TRIF associates with TANK-binding kinase-1 (TBK-1)/IκB kinase-ε (IKK-ε) through NF-κB activating kinase (NAK)-associated protein 1 (NAP1) and tumor necrosis factor (TNF) receptor-associated factor 3 (TRAF3) to phosphorylate IRF3, which then forms a dimer to translocate to the nucleus for IFN-ß expression [32]. For NF-κB activation, TRIF binds to TRAF6 and receptor-interacting protein 1 (RIP1). TRAF6 is an ubiquitin ligase and thus ubiquitinylation of RIP1 by TRAF6 is recognized by TAK (transforming growth factor ß-activating kinase) binding protein 2 (TAB2) and TAB3, leading to activation of TAK1 and then phosphorylation of IKKα and IKKß. These two activated kinases then phosphorylate IκBα which undergoes degradation by ubiquitinylation and releases NF-κB to the nucleus [33]. TAK-1 also activates MAPK (mitogen-activated protein kinase), c-Jun N-terminal kinase, p38, and extracellular signal-regulated kinase, which leads to phosphorylation and activation of the members of the AP-1 family [32], which also triggers IFN-ß gene transcription. 

#### 2.1.2. TLR7/8/9

TLR7/8 recognize dsRNA as short as 19-21 bps in size. TLR7/8 are constitutively expressed at high levels in specific peripheral blood mononuclear cells (PBMCs). TLR7 in human is highly expressed in plasmacytoid dendritic cells (pDCs) whereas TLR8 is mainly found in monocytes [34]. TLR9 senses unmethylated CpG DNA in the genome in DNA viruses, which induces the production of type I IFN in pDCs [35]. All three TLRs are anchored on the endosomal membranes [28]. Different from TLR3 which uses TRIF as a primary adaptor, TLR7/8/9 use MyD88 to induce IFN expression [36]. For TLR7/8, the signaling starts with the MyD88 binding to TRAF6 and IRAK-1 (IL-1 reporter associated kinase 1). This complex leads to the IRF-5 and IRF-7 activation [37]. Similarly, TLR9 also causes MyD88 to interact with IRF7 with the help of a kinase complex containing TRAF6 and IRAK-1 or IRAK-4. For different functions in different cell types, different family members of IRAK are involved. In pDCs, IRAK-1 is required while IRAK-4 is responsible in HEK cells [38]. Interestingly, IRF7 can also interact with either MyD88 or TRAF6 [39]. For IRF7 activation, the IRF7/IRAK-1 complex leads to the IRAK-1-mediated IRF7 phosphorylation. The phosphorylated IRF7 then dimerizes and translocates into the nucleus mainly for IFN-α expression. In this process, IKKα, which is known to participate in the NF-κB activation, is essential. Thus, the IRAK4-IRAK1-IKKα kinase cascades may lead to the IRF7 activation [39]. In summary, the TLR7/8 and TLR9 pathways in the endosome activate IRF7 through MyD88 and account for production of IFN-α in pDCs, which plays a crucial role during virus infection. The TLR3-mediated IRF3 and NF-κB activation through TRIF is an additional scenario to induce IFN-ß gene expression, which may present an alternative role in anti-viral immunity, especially for RNA viruses including PRRSV.

### 2.2. Cytoplasmic Receptors and Their Signaling

#### 2.2.1. RIG-I/MDA5 Pathway

Retinoic acid inducible gene I (RIG-I) is an intracellular receptor for viral dsRNA. It contains a C-terminal DExD/H box-RNA helicase domain and two N-terminal caspase recruitment domains (CARDs) through which RIG-I induces type I IFN. Only RNA molecules that can be efficiently recognized and unwound by the helicase domain of RIG-I have the potential to induce type I IFN through CARD. Therefore, the helicase domain of RIG-I acts as a switch to control whether CARD is revealed and available for activation of downstream processes [34]. Once the CARD domain of RIG-I is uncovered by conformational changes under stimulation, another CARD-containing protein IFN-ß promoter stimulator-1 (IPS-1) is recruited as an adaptor through CARD-CARD interaction. The CARD-CARD interaction between RIG-I and IPS-1 is essential for dsRNA-induced activation of NF-κB, IRF3, and IRF7 [40]. For IRFs activation, IPS-1 transduces signals through TRAF3, a signaling factor involved in the TLR-activation pathway, which in the case of the RIG-I-IRF pathway associates with NEMO and TANK to facilitate the recruitment of TBK1 and IKKε to form the IPS-1-TRAF complex. This complex leads to TBK1/IKKε activation, which then phosphorylates IRF3 and IRF7. Phosphorylated IRF3 and IRF7 form homo- or hetero-dimer and are transported to the nucleus. For NF-κB activation, IPS-1 interacts with TRAF2, TRAF6, and RIP1, which is similar to TLR3-activated NF-κB activation [38]. Once in the nucleus, the active form of IRF3 or IRF7, NF-κB, AP-1, together with other co-transcription factors, such as CREB-binding protein (CBP) form an IFN enhanceosome to produce IFN-ß and IFN-α [39].

An important question is the relative importance of signaling mediated by RIG-I *versus* TLRs. Gene-knockout studies show that RIG-I and TLR pathways mediate different antiviral signaling in different cell types. The ability to induce IFN-ß is severely compromised in RIG-I knockout conventional DCs and fibroblasts, whereas the IFN-ß induction is absolutely normal in RIG-I knockout plasmacytoid DCs, but not in the absence of MyD88 and TRIF [41]. Overall, RIG-I and TLR pathways are not redundant, but rather sense the virus in multiple ways in different cell types so that the host can fight against the virus more efficiently. Besides RIG-I, MDA5 and LPG2 have also been identified as DExD/H box RNA helicase that functions in the antiviral immune response. Like RIG-I, MDA5 contains two N-terminal CARD domains and promote the IFN-ß induction. In contrast, LPG2 lacks the CARD domain and thus is considered as a negative regulator in the RIG-I pathway [42,43].

#### 2.2.2. IPS-1 Signaling

IPS-1 has been proven to play an essential role in the antiviral signaling pathway. Over-expression of IPS-1 leads to activation of both IRF3 and NF-κB and thus type I IFN production. Besides the N–terminal CARD domain, IPS-1 also contains a proline (PRO) region and a C-terminal hydrophobic transmembrane (TM) region that anchors to the mitochondrial outer membrane [37,44]. Mutational analysis shows that the CARD and TM domains are both essential for IPS-1 to retain its function. The mislocalization of IPS-1 greatly impairs the ability to induce IFNs [37], suggesting the involvement of mitochondria in innate immunity. IPS-1 is also able to interact with Fas-associated death domain (FADD) or to directly bind to the C-terminal region of IKKα and IKKβ kinases to activate NF-κB. Both FADD and mitochondria have the ability to induce the apoptosis pathway through recruiting apoptotic factors and releasing pro-apoptotic proteins, respectively. This hints that apoptosis may be one of the innate immune responses and IPS-1 may act as a bridge between apoptosis and type I IFN response.

#### 2.2.3. RNA Recognition by RIG-I and MDA5

RIG-I and MDA5 are cytoplasmic receptors to recognize RNA. They are structurally similar, but functionally different. Studies show that MDA5 is essential for the recognition of picornaviruses and poly(I:C) [45,46]. RIG-I is required for the recognition of many negative-strand RNA viruses including Sendai virus and influenza A virus, and poly(I:C) is often used to stimulate the RIG–I/MDA5 pathway [46]. Compared to MDA5, RIG-I senses RNA transcribed *in vitro* by polymerases. Actually, both single- and double-strand RNA with a triphosphate moiety at the 5' end can activate type I IFN production via RIG-I [47,48]. This leads to a crucial question ‘How the host or such cytoplasmic receptors distinguish viral RNAs from self-RNAs’. The answer is not hard but complicated. Cellular RNAs like ribosomal RNA (rRNA) and transfer RNA (tRNA) possess a 5' monophosphate instead of 5' triphosphate and thus would not be detected by RIG-I. For messenger RNA (mRNA) and small nuclear RNAs (snRNAs) however, the 5' end is modified with a methyl-guanosine cap which protects RNAs from degradation. In addition, cellular RNAs are often post-transcriptionally modified by pseudouridine, 2-thiouridine, or 2'-*O*-methylation, and these modifications suppress the immune-stimulatory activity of RNA even if the RNA contains a 5' triphosphate [33,48]. Last but not least, cellular RNA generally does not exist as “naked” molecules in a cellular environment but rather forms complexes with a multitude of RNA-binding proteins. Thus, it is conceivable that protein components of the complexes may also play a role in marking certain RNA species as ‘self’ [33].

### 2.3. IFN Signaling Pathways

After the first wave of IFN-ß production, the response enters into the second stage, where hundreds of genes are upregulated and the resulting proteins are involved in a variety of functions. These genes are referred to as IFN-stimulated genes (ISGs), and the pathway that transduces the IFN signal for expression of ISGs is the JAK-STAT pathway.

#### 2.3.1. JAK-STAT Pathway

The JAK-STAT signaling pathway is initiated when IFN-α and IFN-ß bind to their receptors on the cell surface. The receptor binding activates Janus kinase (JAK) and Tyk2 to phosphorylate the signal transducers and activators of transcription 1 (STAT1) and STAT2. Phosphorylated STAT1 and STAT2 form heterodimers which associates with IRF9 to form the interferon-stimulated gene factor 3 (ISGF3) complex. The ISGF3 complex is imported into the nucleus and binds to IFN stimulated response element (ISRE) to induce ISGs resulting in the establishment of an antiviral state. Once the ISGF3 complex translocates to the nucleus, both phosphorylated STAT1 and STAT2 undergo dephosphorylation and are redistributed back to the cytoplasm [49].

The JAK-STAT pathway is regulated by feedback inhibitors of three STAT negative regulators. The suppressor of cytokine signaling (SOCS) family suppresses the phosphorylation of STATs by inhibiting the association of JAK with the IFN receptors [50]. The protein inhibitor of activated STATs (PIAS) family consists of PIAS1, PIAS3, and PIASy. PIAS1 and PIAS3 compete against STAT1 and STAT3 for ISRE within the promoter region, respectively, and thus suppress the expression of ISGs [51]. PIASy can recruit other factors to repress the transcription activities of STAT1. In addition to the negative regulation of STATs, PIAS is also identified as a SUMO E3 ligase. SUMO is a small ubiquitin-like modifier and many proteins are post-translationally modified by SUMO, which is a process known as sumoylation. Sumoylation occurs similarly to ubiquitinylation in such a way that a SUMO molecule is conjugated to its target protein through specific sequence recognition by E3 ligase [52]. Sumoylation plays roles in diverse biological processes including regulation of gene transcription, chromatin structure, protein localization and functions. SUMO E3 ligase is required for ubiquitinylation of certain proteins and mediates degradation.

#### 2.3.2. IFN Inducible Genes and Their Functions

More than 300 ISGs have been identified so far but a relatively few of these ISGs have been implicated in instigating the antiviral state including catalysis of cytoskeletal remodeling for apoptosis, regulation of post-transcriptional events, and post-translational modifications. Many other ISGs function as pattern-recognition receptors (PRRs) to sense viral molecules or encode transcription factors that participate in the amplification loop increasing IFN production and preventing dissemination of virus [53]. Among all ISGs, ISG15, GTPase, myxovirus resistance A (MxA), ribonuclease L (RNaseL), and protein kinase K (PKR) are the most extensively studied, and function as antiviral effectors.

*ISG15:* ISG15 is an ubiquitin homolog [54]. Since ubiquitylation is involved in innate immune signaling, ISGylation mediated by ISG15 is thought to have IFN regulatory function. ISG15 is expressed as a 165-amino acid protein and processed to expose the C-terminal LRLRGG sequence. When this sequence is exposed in the case of ubiquitylation, ubiquitin is conjugated to its substrate by three enzymes; ubiquitin-activating enzyme (E1), ubiquitin-conjugating enzyme (E2), and ubiquitin ligase enzyme (E3) [55]. For ISGylation, ISG15 is conjugated to its substrates by similar to or even the same enzyme. The E1-like ubiquitin-activating enzyme is specific for ISG15, whereas E2 ubiquitin-conjugating enzyme and E3 ubiquitin ligase possess the ability to conjugate and ligate both ubiquitin and ISG15. At least 158 putative ISG15 substrates have been identified and many of them play important roles for type I IFN responses including JAK1, STAT1, RIG-I, Mx1, and PKR functions [53]. Similar to ubiquitylation, ISGylation is also reversible. However, unlike the Lys48-linked ubiquitin leading to proteasomal degradation, ISGylation parallels the K63-linked ubiquitin to recruit other proteins and modulates the function of enzymes. ISGylation can prevent virus-mediated IRF3 degradation [56]. ISG15 may also be secreted outside cells in a large amount and act as a cytokine to modulate immune responses [57].

*Mx:* Type I IFNs induce the expression of several quinine-hydrolysis proteins. The Mx family of proteins belongs to this class of proteins involved in mediating vesicle budding, organogenesis, and cytokinesis, but only MxA in humans has been shown to have an antiviral activity [58,59]. The Mx proteins have a large N-terminal GTPase domain, a center interacting domain (CID), and a C-terminal leucine zipper (LZ) domain. Both the CID and LZ domains are required to recognize target viral structures mostly nucleocapsid-like structures [60]. In normal circumstances, the MxA protein accumulates on the intracellular membrane and forms oligomers by association between the LZ and CID domains. During virus infection, MxA monomers are released and bind to viral nucleocapsids or other viral components and trap them for degradation.

*OAS1:* 2', 5'-oligoadenylate synthetase 1 (OAS1) is constitutively expressed in cells at low levels and upregulated by type I IFNs. OAS1 resides in the cytoplasm as an inactive monomer. Once activated by viral RNA, this enzyme oligomerizes and forms a tetramer which synthesizes 2′-5′ linked oligoadenylates (2-5A). The binding of 2-5A to RNase L triggers the dimerization of RNase L enabling it to cleave cellular and viral RNAs. The cleaved RNAs then activate cytoplasmic PRRs such as RIG-I and MDA5 [53,61].

*PKR: *PKR belongs to a family of protein kinases that respond to environmental stresses and regulate protein syntheses [62]. PKR is constitutively expressed in all tissues at basal levels and up–regulated by type I and type III IFNs [63]. Under normal circumstance, PKR remains as an inactive monomer. During infection, viral RNA elicits a conformational change of PKR and allows ATP to bind to its C-terminal kinase domain for activation. Activated PKR dimerizes and phosphorylates eukaryotic initiation factor 2α (EIF2α) to halt the translational process [53]. Two RNA-binding motifs (RBMs) are found in the N-terminal region of PKR, and these motifs bind RNA by recognizing a specific higher ordered structure. Longer RNA moieties engage both RBMs of PKR and activate the kinase function, although RNAs binding to PKR need only be 16 bps long [64]. Consequently, dsRNAs longer than 30 bps activate PKR efficiently and ssRNA of 47 bases with a 5'-triphophate have a limited ability to active PKR [65]. 

In general, ISG15 and MxA are expressed only after IFN stimulation, whereas OAS-RNaseL and PKR are expressed constitutively at low levels but increased by type I IFN stimulation. Constitutive expressions of OAS and PKR suggest that these proteins act not only as effectors but also as dsRNA-specific PRRs that can trigger antiviral responses.

## 3. Modulation of Innate Immunity by PRRSV

In PRRSV-infected swine, the virus persists through a ‘smoldering’ type of infection in which the virus continuously replicates at a low level. The virus first develops an acute infection lasting for 3 to 4 weeks followed by persistent infection in some animals. During persistent infection, viremia is absent and viral replication sites are restricted primarily to lymphoid tissues and other immune-privileged sites such as tonsils and lymph nodes [66,67]. Persistence of PRRSV is also common in the semen of infected boars [68]. Virus shedding from persistently infected pigs is an important source for transmission and is one of the main factors that hinder the control of PRRSV infection in the field. Viral persistence may be the result of a poor immune response, including delayed production of neutralizing antibodies and suppression of innate immune responses.

**Figure 2:**
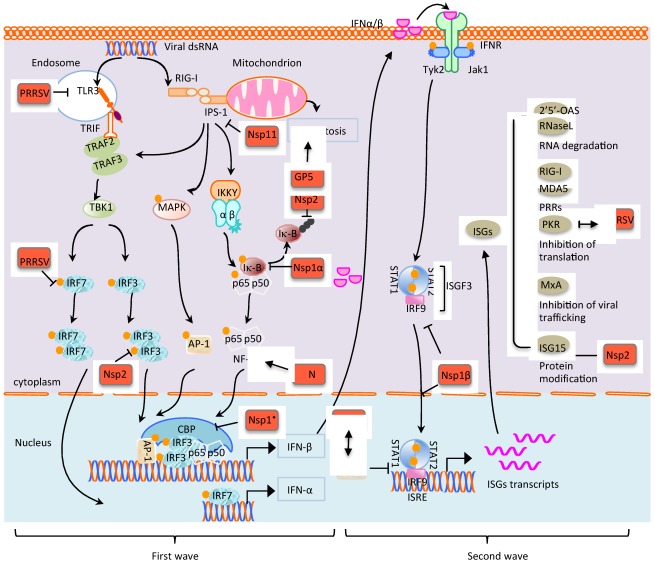
The modulation of type I IFN production by PRRSV. PRRSV has been shown to reduce the expression of TLR3 and IRF7 in pDCs. While Nsp1 has the ability to degrade CBP in the nucleus, Nsp1α subunit inhibits the IκB phosphorylation in the cytoplasm. Nsp2 inhibits IFN production by blocking the ubiquitinylation of phosphorylated IκB and phosphorylation of IRF3 through the OTU domain. Nsp11 suppresses IFN-β production through degradation of IPS-1 mRNA. For the second wave of IFN signaling, PRRSV Nsp1β blocks the phosphorylation of STATs and inhibits the nuclear translocation of ISGF3 complex. Nsp1 interacts with PIAS. Since PIAS is a multi-functional protein, Nsp1 interaction may also modulate pathways other than the JAK-STAT pathway. PKR is redistributed during PRRSV infection. Nsp2 has the potential to deconjugate ISGylation. The GP5 protein induces apoptosis. Since Nsp1 is cotranslationally processed to Nsp1α and Nsp1β in PRRSV-infected cells, the uncleaved form of Nsp1 is unlikely to exist in virus-infected cells. It is unknown which subunit of Nsp1 interacts with PIAS and degrades CBP.

Recent studies have described cytokine responses during PRRSV infection. In PRRSV-infected pigs, TNF-α is down-regulated while IL-10 production is up-regulated. Pigs do not show typical innate immune responses including type I IFN response, although PRRSV is highly susceptible to IFN-α/β and its growth is inhibited. PRRSV-infected pigs display low levels of IFN-α despite the abundant presence of viral RNA in infected cells. IFN-α is not detectable in the lungs of virus-infected pigs where PRRSV actively replicates [69,70]. The suppression of IFN production is also evident in virus–infected MARC-145 and PAM (porcine alveolar macrophage) cells [69,71,72], suggesting that PRRSV suppresses IFN production. Such inhibition by PRRSV is RIG-I mediated [73]. In pigs infected with the newly emerged highly pathogenic PRRSV (HP-PRRSV), the virus replicates rapidly and readily persists. RNA microarray analysis of HP-PRRSV-infected cells shows that production of swine type I IFNs are suppressed, suggesting that the host innate immune response is blocked [5]. The European genotype of PRRSV has also been examined by microarray analysis for its ability to modulate global gene expression [74]. A repression of IFN-α transcription and a delay of IFN-ß transcript accumulation in infected-microphages was shown. Here, we discuss below the manipulation of IFN signaling pathways by PRRSV during infection (Figure 2).

### 3.1. TLRs and the Virus

Although a large body of information is available for viral modulation of TLRs for humans, porcine TLRs are relatively poorly understood. TLR9 is expressed in Peyer’s patches in pigs, and its expression is also stimulated in monocyte-derived dendritic cells [75]. Porcine TLR3 and TLR7 seem to be important for PRRSV [76,77], and porcine TLR8 has also been identified. Porcine TLR3 is highly expressed in kidney, duodenum, spleen, and liver, whereas porcine TLR7 is moderately expressed in bone marrows, intestines, spleen, livers, lungs, mesenteric lymph nodes, trachea, thymus, kidneys, and skin [77]. Differential expressions of TLRs have been studied for PAMs and porcine bone marrow hematopoietic cell-derived immature dendritic cells (imDCs) in response to poly(I:C) and PRRSV [78]. In unstimulated cells, expression levels of TLR3, TLR7, and TLR8 were higher in PAMs than in imDCs. Poly(I:C) tends to stimulate the TLR3 expression in PAMs but inhibits TLR7 and TLR8 expression. In contrast, PRRSV down-regulates TLR3, TLR7, and TLR8 expression levels at 6 h post-infection (p.i.) in imDCs, which return to basal levels by 24 h p.i.. Interestingly, TLR7 expression decreased continuously through 24 h of infection. In other study, the TLR3 activation caused increased antiviral response to PRRSV and decreased viral replication [76]. TIR-domain truncated TLR3 was found to increase the PRRSV infection in MARC-145 cells, while wild-type TLR3 inhibited PRRSV replication. Thus, TLR3 seems to play an important role in establishing effective innate immunity to PRRSV. Another study [79] using poly(I:C) as a TLR3-specific agonist and LPS as a TLR4-specific agonist showed that PRRSV infection increased IFN-α expression at 16 h p.i. in PAMs, and this was an 8 h delay compared to poly(I:C) stimulation alone. No significant increase of IFN-α was observed upon infection [79]. When PRRSV-infected cells are stimulated by poly(I:C) or LPS, TLR4 was not influenced but TLR3 was significantly reduced, and the TLR3 down–regulation facilitated virus replication.

### 3.2. PRRSV Targeting IFN Enhanceosome through IRF-Mediated RIG-I/MDA5 Pathways

The abundance of IFN-α and IFN-ß transcripts are unaffected by PRRSV infection in MARC-145 cells [72], suggesting that PRRSV infection does not result in the induction of type I IFN production. The mRNA levels for IFN and enhanceosome genes were enhanced following tRNA treatment. However, during concurrent tRNA treatment and PRRSV infection, IFN-α, IFN-ß, ATF-2, and IRF3 (but not NF-κB and JUN), were significantly reduced relative to tRNA treatment only [72], and this virus-mediated inhibition was via the RIG-I signaling pathway [73]. PRRSV activates NF-κB and AP–1 but inhibits IRF3 which is mapped upstream of RIG-I. 

PRRSV Nsp’s have been screened for type I IFN suppression, and four Nsp’s were identified to contain the suppressive activities: Nsp1, Nsp2, Nsp4, and Nsp11 [80]. Nsp1 contains the strongest potential to inhibit the IFN-ß promoter activity, and both Nsp11 and Nsp1ß have the ability to inhibit IRF3-mediated type I IFN production. The phosphorylation and nuclear translocation of IRF3 are inhibited by Nsp1ß. The mechanism for Nsp1-mediated IFN inhibition is partly due to the degradation of CREB (cyclic AMP responsive element-binding)-binding protein (CBP) in the nucleus [80], which disrupts the formation of enhanceosome crucial for IFN gene expression. The CBP degradation is proteasome-dependent but independent from the PCP activity of Nsp1 (Figure 2). 

Nsp2 also down-regulates type I IFN induction (Figure 2). Nsp2 is a membrane-anchored protein that contains a CP activity and a deconjugation activity associated with ovarian tumor (OTU) domain-containing protease activity. While the deconjugation activity is suggested to function in ISGs and NF–κB signaling, the CP activity inhibits IRF3 phosphorylation and nuclear translocation [81]. The mechanism for the CP mediated-IRF3 deactivation is unknown.

Nsp11 participates in the suppression of RIG-I signaling. Different from Nsp1 which is localized in the cytoplasm and the nucleus, Nsp11 expression is mainly cytoplasmic suggesting its main function is in the cytoplasm. Nsp11 contains a domain designated the NendoU domain and functions as an endoribonuclease [82]. The NendoU domain consists of two subdomains; subdomain A and subdomain B. Subdomain A contains active sites for endoribonuclease activity, while subdomain B is thought to maintain the overall structure of Nsp11. We have been studying the role of Nsp11 for IFN regulation and have found that Nsp11 blocks the phosphorylation of both IRF3 and IκB. This leads to the inhibition of the nuclear translocation of IRF3 and NF-κB, and as consequences, IFN production is inhibited. IPS-1 is an upstream component in the RIG-I pathway and is found to be degraded in Nsp11-expressing cells and PRRSV-infected cells (Figure 2). The IPS-1 degradation results in the inhibition of down-stream pathways which is likely the basis for suppression of IRF3 and NF-κB activation by Nsp11. Using a series of NendoU mutants, the relationship between IPS-1 degradation and NendoU activities was examined and the results indicate that the Nsp11 endoribonuclease activity is associated with the degradation of IPS-1 mRNA.

### 3.3. Modulation of NF-κB Signaling by PRRSV

#### 3.3.1. Up-regulation of NF-κB by N protein

Viruses may activate or inhibit the NF-κB signaling for their own benefits. The NF-κB signaling pathway may function as a protective response of the host to a virus, and thus, many viruses have evolved to block NF-κB activation to evade host innate immunity. In this scenario, NF-κB signaling is inhibited by PRRSV which suppresses a type I IFN response. However, a study shows that NF-κB may be up-regulated by PRRSV at 36–48 h p.i. [83]. The NF-κB activation during this time of infection was dose-dependent and replication-dependent since UV-inactivated PRRSV did not up-regulate the response. In porcine pDCs, PRRSV promotes NF-κB phosphorylation when stimulated with transmissible gastroenteritis virus (TGEV) [84]. The data show that PRRSV actually activates the NF–κB pathway in MARC-145 cells and PAMs at a late stage of infection. Activation of the NF-κB pathway by viruses may optimize viral replication and control of host cell proliferations and apoptosis. However, blocking NF-κB activation by over-expressing the dominant negative form of IκBα did not alter viral replication [73,83], suggesting that the activation of NF-κB by PRRSV may be involved in other cellular progresses such as apoptosis or modulation of cytokines.

The PRRSV N protein appears to stimulate NF-κB activation and the activation is dose-dependent (Figure 2). The region at amino acid positions 30–73 of N is responsible for NF-κB activation, and within this region, both the nuclear localization signal (NLS) and nucleolar localization signal (NoLS) are located. Thus, it is plausible that NF-κB activation may be linked to the N protein nuclear localization. The region is also important for homo-dimerization of N, which is embedded in the NF–κB activation domain. This implies that N-N homo-dimerization may be associated with the NF–κB activation.

#### 3.3.2. Down-Regulation of NF-κB by Nsp1α and Nsp2

During the course of our study, Nsp1α was found to be able to inhibit the activation of NF-κB [85]. This finding differs from the previous report [83]. Using the NF-κB and PRDII luciferase reporter constructs, Nsp1α was determined to reduce the luciferase activities of both reporters 24 h post–transfection. In the presence of Nsp1α, the phosphorylation of IκBα was inhibited, leading to the block of NF-κB nuclear translocation upon stimulation with tumor necrosis factor α (TNFα). Nsp1α is the N-terminal subunit of Nsp1 and contains the PCPα protease domain. A zinc finger motif is formed by four catalytic residues of histidine (H) or cysteine (C) to hold a zinc ion and is commonly found in transcription factors. PRRSV Nsp1α contains two zinc finger motifs; one motif (ZF1) of C8-C10-C25-H28 and another motif (ZF2) of C70-C76-H146-M180. After cleavage, the PCPα activity is self–inactivated by conformational changes and thus is less likely responsible for suppression of the IFN response. This suggests that two zinc finger motifs may be associated with Nsp1α-mediated IFN suppression. A deletion mutant of Nsp1α lacking the C-terminal 14 amino acids, in which the second zinc finger motif is lost, shows no inhibition of IFN induction. This finding raises a possibility that zinc finger motifs of Nsp1α may take a part in the suppression of NF-κB or other type I IFN pathways. Using a series of mutant constructs, the first zinc finger motif has been mapped to be important for IFN regulation since the mutants containing the first zinc finger motif catalytic sites always reverse the IFN suppression [86]. Although it is unclear whether the CBP degradation by Nsp1 is mediated by Nsp1α or Nsp1β, it is possible that the cytosolic form of Nsp1 blocks the NF-κB activation and the nuclear form of Nsp1 degrades CBP possibly through the zinc finger domain.

The OTU domain of Nsp2 is able to deconjugate the ubiquitin or ISG15 from substrates [87]. Since ubiquitinylation is involved in the RIG-I and TLRs signaling such as the recruitment of adaptor proteins and degradation of IκBα, Nsp2 likely inhibits the type I IFN production via its OTU domain. Indeed, Nsp2 has been shown to be able to down-regulate the NF-κB activity although its mechanism is unknown [81]. Sun *et al*. [88] have reported that Nsp2 may interfere with polyubiquitinylation of phosphorylated IκB, resulting in the failure of IκB degradation. Further studies suggest that NF-κB may be down-regulated by Nsp’s at the early stage of virus infection.

### 3.4. Inhibition of JAK-STAT Signaling and ISG Expressions by PRRSV

PRRSV suppresses the type I IFN signaling not only during the production of IFNs but also after IFN secretion. In IFN-α-induced MARC-145 cells, the JAK-STAT pathway is suppressed at 24 h post–infection when using ISG15 and ISG56 mRNAs as indicators. The ISGF3 nuclear translocation is blocked by PRRSV [89]. In TGEV-stimulated pDCs, the nuclear translocation of STAT1 is inhibited by PRRSV [84], and Nsp1ß is the viral protein responsible for this inhibition. Nsp1ß, not Nsp1α, inhibits phosphorylation of STAT1 and nuclear translocation of ISGF3 [89,90]. In our laboratory by yeast-two-hybrid assays, Nsp1α has been found to interact with PIAS1 [91]. PIAS functions as a negative regulator for STAT1 and also as the SUMO E3 ligase. The interaction of Nsp1α with PIAS1 may lead to the Nsp1α sumoylation and facilitate the Nsp1α transport into the nucleus. Besides, degradation of CBP by Nsp1 may also require the E3 ligase activity of PIAS. Nsp1 may bring CBP to PIAS1 and then CBP is sumoylated by PIAS1, which facilitates the ubiquitinylation of CBP for degradation.

Less is known about the effects of PRRSV infection on ISGs. In MARC-145 cells, anti-PRRSV activity of type I IFNs is highly correlated with the MxA promoter activity and less correlated with IRF3 and IRF7 induction [25]. This suggests that MxA would be a better biomarker to monitor the IFN induction during PRRSV infection than other ISGs. ISG15 is an ubiquitin-like molecule and can be reversibly conjugated to proteins to mediate important innate antiviral responses. The OTU domain in Nsp2 of PRRSV contains a deubiquinylation activity. Nsp2 can decrease ubiquitinylation and ISGylation in 293T cells, indirectly indicating that Nsp2 may help the virus evade the innate immune response by deconjugation [87]. Typically, PKR is found in the both cytoplasmic and perinuclear regions in cell, but its distribution is altered by increased signals in the perinuclear region and within the nucleoli of PRRSV-infected cells. This localization pattern of PKR is similar to that of PRRSV N protein but N and PKR are not co-localized in the nucleoli [92]. 

Combining the information, it seems that in the early stage of PRRSV infection, Nsp1, Nsp2, and Nsp11 facilitate viral gene transcription and viral protein translation, while suppressing the immune responses of infected cells by inhibiting type I IFN production and IFN-inducible signaling pathways. At 36 to 48 h p.i., when virus infection enters to the late stage, the NF-kB pathways are activated to assist the survival of virus in the host.

### 3.5. Apoptosis and the Virus

The autocrine and paracrine actions of type I IFNs and other cytokines encompass the comprehensive host immune response to defend against viral infection. This is accompanied by a reciprocal activation of programmed cell death in virus-infected cells and the reduction of viral load in the host. Apoptotic cells are observed in PRRSV-infected tissues of pigs [93], while in other studies, apoptotic cells are not PRRSV-infected [94,95]. Based on the different observations, some researchers have reported that the GP5 protein expression induced apoptosis by acting downstream of B cell lymphoma-2 (Bcl-2) and mapped the apoptotic region to the N-terminal 119 amino acids of GP5 [96]. Other researchers however propose that ‘bystander’ cells undergo apoptosis during PRRSV infection supported by their microarray data in MARC-145 cells indicating that only minimal increases of pro–apoptotic gene transcripts in PRRSV-infected cells compared to uninfected cells [97]. Later, studies using porcine alveolar microphages [98] and monocyte-driven dendritic cells [99] reveal that it is the stage of virus infection that determines whether PRRSV-infected cells are towards anti-apoptosis or pro-apoptosis. At 8 h post-infection, the balance gears towards anti-apoptosis, but all infected cells will eventually die by both apoptosis and necrosis. The expression of viral proteins is necessary to induce a strong anti-apoptotic state and it is possible that non-structural proteins are involved in the apoptotic suppression since the PRRSV-mediated anti-apoptosis effect was already present before structural proteins were detectable in infected microphages [98]. As discussed earlier, both type I IFN production and virus-induced apoptosis are important defense mechanisms of a host against invading viruses and they both share common pathways. Therefore, it is possible that PRRSV may actively interfere with the induction of apoptosis, which may be partially due to its ability to inhibit type I IFN production, especially in the early stage of infection.

IPS-1 interference is a good suggestion for PRRSV-mediated apoptosis. IPS-1 can induce the RIG-I signaling pathway, leading to the expression of type I IFNs and the mitochondria-mediated apoptosis. Studies indicate that IPS-1 functions as a bridge between the type I IFN induction and apoptosis. Nsp11 is able to degrade IPS-1 mRNA and results in the suppression of type I IFN induction. It is unknown whether the degradation of IPS-1 will also inhibit the cellular apoptosis. Nsp15 of severe acute respiratory syndrome coronavirus (SARS-CoV), which is the endoribonuclease homolog of PRRSV Nsp11, suppresses the IPS-1 induced apoptosis independent from IRF3 activation [100], implying that the IPS-1 suppression may lead to inhibition of apoptosis as a method of immune evasion for PRRSV.

## 4. Conclusion and Future Prospects

Innate immunity, especially the type I IFN signaling, is the first line defense of host cells to fight against virus infection. In the present review, multiple strategies of PRRSV for manipulating TLR and RIG-I-mediated IFN production and JAK-STAT signaling pathways are discussed. During the intricate signaling processes, other pathways including the NF-κB pathway are activated or inhibited by PRRSV. The N protein appears to activate the NF-κB pathway and its nuclear localization and dimerization may be associated. Nsp1, Nsp2, and Nsp11 are identified to be responsible for viral immune-modulation and their domains and motifs for biological mechanisms have partially been studied at the molecular level. Nsp1 interferes with the IRF3-mediated IFN production in the nucleus and the NF-κB-mediated pathway in the cytoplasm. For IFN signaling, the Nsp1ß subunit of Nsp1 suppresses the JAK-STAT pathway by blocking different steps in different cell types. Besides, Nsp1 (Nsp1α subunit or Nsp1β subunit, or both) interacts with PIAS1. Since PIAS is a nuclear protein with multiple functions, the interaction with PIAS opens a new direction of the host cellular modulation by Nsp1, which may or may not relate to IFN pathways. Nsp2 is involved in the blocking of IRF3 activation through the activity of PCP2 and reduces ubiquitinylation and ISGylation through the OTU domain. Nsp11 degrades IPS-1 mRNA via the NendoU domain and impedes the downstream IRF3 and NF-κB activation. Despite the progress made on the PRRSV-mediated modulation of innate immune responses, studies have mainly been conducted in MARC-145 which are cells derived from monkey kidney, or in PRRSV non-permissive cells such as HeLa, raising questions as to their relevance to natural infection. Some limited studies have been conducted in PAMs as the natural target cells and pigs as the natural host animal. Different PRRSV isolates differ in their sensitivity to IFN-α and they also seem to have different abilities to down-regulate the IFN response [101]. Marked differences have been observed in different cell types for IFN-ß induction [102]. A vaccine strain of PRRSV does not exhibit sensitivity to IFN in MARC-145 cells, whereas both virulent PRRSV and the vaccine strain are sensitive to IFN in PAMs. The PKR inhibitor 2-aminopurine (2-AP) restored the virus replication in MARC-145 cells but did not rescue the virus in PAMs. In addition to different cell types and various virus isolates, different genetic breeds of pigs may yield different responses. PAMs from Landrace pigs inhibit or delay the PRRSV growth compared to those from other breeds of pigs [103]. The lack of knowledge on the mechanistic basis for how PRRSV evades host immune responses leads to an inadequate control of the disease. One of the key questions is whether the virulence of PRRSV is linked to immunosuppression. Identification of active sites for IFN modulation and generation of mutant PRRSV using infectious cDNA clones to eliminate the immune suppressive functions from the virus may be feasible ways to generate new and attenuated vaccines for PRRS.
